# Genome-wide expression profile of first trimester villous and extravillous human trophoblast cells

**DOI:** 10.1016/j.placenta.2010.10.010

**Published:** 2011-01

**Authors:** R. Apps, A. Sharkey, L. Gardner, V. Male, M. Trotter, N. Miller, R. North, S. Founds, A. Moffett

**Affiliations:** a Department of Pathology and Centre for Trophoblast Research, University of Cambridge, Tennis Court Road, Cambridge CB2 1QP, UK; b Laboratory for Regenerative Medicine, University of Cambridge, UK; c Division of Reproduction and Endocrinology, St. Thomas’ Hospital, London, UK; d School of Nursing, University of Pittsburgh, United States

**Keywords:** Trophoblast differentiation, Microarray, LAIR-2, Uterine NK cells, First trimester, VT, villous trophoblast, EVT, extravillous trophoblast, uNK, uterine NK, ECM, extracellular matrix

## Abstract

We have examined the transcriptional changes associated with differentiation from villous to extravillous trophoblast using a whole genome microarray. Villous trophoblast (VT) is in contact with maternal blood and mediates nutrient exchange whereas extravillous trophoblast (EVT) invades the decidua and remodels uterine arteries. Using highly purified first trimester trophoblast we identified over 3000 transcripts that are differentially expressed. Many of these transcripts represent novel functions and pathways that show co-ordinated up-regulation in VT or EVT. In addition we identify new players in established functions such as migration, immune modulation and cytokine or angiogenic factor secretion by EVT. The transition from VT to EVT is also characterised by alterations in transcription factors such as STAT4 and IRF9, which may co-ordinate these changes. Transcripts encoding several members of the immunoglobulin-superfamily, which are normally expressed on leukocytes, were highly transcribed in EVT but not expressed as protein, indicating specific control of translation in EVT. Interactions of trophoblast with decidual leukocytes are involved in regulating EVT invasion. We show that decidual T-cells, macrophages and NK cells express the inhibitory collagen receptor LAIR-1 and that EVT secrete LAIR-2, which can block this interaction. This represents a new mechanism by which EVT can modulate leukocyte function in the decidua. Since LAIR-2 is detectable in the urine of pregnant, but not non-pregnant women, trophoblast-derived LAIR-2 may also have systemic effects during pregnancy.

## 1 Introduction

In the early weeks of human pregnancy the embryo implants completely into the uterine wall, surrounded by trophoblast cells. Trophoblast progenitors located at the basement membrane of the placental villi differentiate into distinct lineages with specialized functions [1]. Villous trophoblast (VT) cells proliferate and cover the mesenchyme and fetal vessels that form the placental villous tree. Subsequent fusion of VT generates an overlying syncytium of villous syncytiotrophoblast, which provides a large surface area for nutrient and gas exchange as well as synthesis of hormones such as progesterone. A distinct lineage of extravillous trophoblast (EVT) cells arises from VT cells in trophoblast columns at the tip of villi anchoring the placenta to the uterine wall. EVT do not divide but, during the first trimester of pregnancy, they detach from the placental villi and invade directly through the decidual stroma as individual cells. These interstitial EVT can penetrate as far as the inner third of the myometrium before forming sessile, multinucleated giant cells. Endovascular EVT advance along the luminal endothelium of decidual spiral arteries, against the direction of flow. EVT invasion is associated with dramatic remodeling of decidual spiral arteries. Interstitial EVT destroy the muscular coat and endovascular EVT displace endothelial cells. Together these trophoblast cells convert the spiral arteries into distensible low-resistance channels to increase blood flow to the growing feto-placental unit. Failure to transform arteries is associated with poor trophoblast invasion and results in inadequate placental perfusion [2]. Clinically, this may present as pre-eclampsia, fetal growth restriction or recurrent miscarriage. Understanding the factors controlling the differentiation of VT to EVT and the extent of EVT migration is therefore crucial if we are to improve the diagnosis and management of these complications of pregnancy.

The factors that control exit of VT from the cell cycle, differentiation into motile EVT and the extent of migration are unclear. The spontaneous differentiation of VT to EVT cells when cultured *in vitro*, strongly suggests that this process is largely controlled by an intrinsic differentiation program. However, interactions with extracellular matrix (ECM), growth factors secreted by cells in the decidua and even oxygen tension also influence trophoblast differentiation [3]. Differentiation of VT to EVT is accompanied by complex changes in phenotype that include the expression of adhesion molecules, such as integrins, metalloproteases and cathepsins which permit EVT migration. Conversely, decidual cells express tissue inhibitors of metalloproteinases (TIMPs) and plasminogen activator inhibitor (PAI) which function to limit invasion. How these interactions are co-ordinated has until recently been unclear. There is now strong evidence that uterine NK (uNK) cells, the dominant lymphocyte in the decidua during early pregnancy, also play a role in regulating trophoblast invasion and vascular conversion [4]. Unlike VT, which lack surface HLA class I or class II, migrating EVT express a unique repertoire of HLA class I: HLA-C, HLA-E and HLA-G. Corresponding receptors for these molecules are present on uNK cells and decidual macrophages at the implantation site [2]. Immunogenetic data suggests that certain combinations of genes from the Killer Immunogobulin-like Receptor (KIR) family expressed on maternal uNK cells and their HLA-C ligands on trophoblast, are associated with an increased risk of pre-eclampsia or recurrent miscarriage [5,6]. Although recognition of trophoblast MHC by receptors such as KIR, LILR and NKG2/CD94 on uNK cells is clearly important, activating and inhibitory receptors that recognise non-MHC self molecules also regulate NK cell functions and cytokine secretion. NK cells integrate the signals from receptors for MHC and non-MHC ligands and responses stimulated by non-MHC receptors can modify those induced by the conventional MHC receptors. Over 30 potential receptor/ligand pairs have been described and uNK cells have been shown to express many of these [7,8]. Understanding of their role in trophoblast/uNK interactions is limited because a systematic screen for known and novel ligands expressed by EVT, has not yet been undertaken.

Efforts to define how VT to EVT differentiation and subsequent migration is controlled have also been hampered by the difficulty in obtaining primary VT and EVT cells from the first trimester. Here we present a microarray analysis of the transcriptome in primary VT and EVT cells, isolated from healthy first trimester pregnancies to a high degree of purity. The aim of this whole genome analysis was to define what transcriptional changes underlie the transition from the villous to the extravillous phenotype. We also examined the expression by trophoblast of potential ligands for receptors on NK cells and other decidual leukocytes to better understand the interactions between EVT and the maternal immune system. Microarrays of the JAR and JEG-3 choriocarcinoma cell lines are also included, to characterise the validity of these models for VT and EVT, since they are frequently used due to the inaccessibility of primary trophoblast cells.

## 2 Methods

### 2.1 Clinical samples and cell lines

Decidual and placental tissues were obtained from elective terminations of normal pregnancies between 8 and 12 weeks gestation. Ethical approval for the use of these tissues was obtained from the Cambridge Local Research Ethics Committee. For histological staining, blocks of tissue were frozen and 5 μm serial sections cut before fixing in acetone and storage at −20 °C. For flow cytometry, single cell preparations of trophoblast or leukocytes were isolated as previously described [9]. Briefly, placental villi were identified macroscopically, digested with trypsin and collected by density gradient to obtain cells which are 50–80% EGF-R+. VT is the only cell type at the implantation site that expresses EGF-R [10]. EGF-R+ cells representing VT were obtained at high purity by staining these trophoblast cells immediately, followed by flow cytometry sorting. Alternatively, the primary trophoblast cells were cultured for 12 h on fibronectin in Hams F12 with 20% fetal calf serum, after which 50–80% of the cells express HLA-G, a marker specific for EVT [9,11]. The resulting HLA-G positive EVT cells were then analysed or purified by flow cytometry as described below. Leukocytes were isolated by collagenase digestion of maternal decidual tissue and stained for flow cytometry immediately. Villous mesenchyme or decidual stromal cells were obtained by at least 4 passages of the adherent placental or decidual cells isolated. Peripheral blood leukocytes were isolated on Lymphoprep (Axis-Shield) from fresh venous blood of normal adult volunteers. Urine was obtained with informed consent from healthy local donors who were not pregnant, or throughout gestation, and were stored at −20 °C until use. Urine from pre-eclamptic subjects was provided by the Pregnancy Exposures and Preeclampsia Prevention (PEPP) study from women at 10 weeks gestation (11 of whom went on to develop pre-eclampsia and 11 healthy controls) and by the SCOPE project from women at 15(±1) weeks gestation (20 of whom went on to develop pre-eclampsia and 20 healthy controls). Both studies were approved by Ethical Board review. JAR and JEG-3 cells were purchased from the American Type Culture Collection and cultured according to their instructions (ATCC reference numbers HTB-144 and HTB-36).

### 2.2 Monoclonal antibodies

Monoclonal antibodies (mAbs) used which bind HLA-G were G233 [12] made in our own laboratory and MEM-G/9-FITC [13] from Serotec. Additional unconjugated mAbs were to IL-15Rα (clone JM7A4) from BioLegend, cytokeratin (clone PKK-1) from Labsystems, IL-2Rβ (clone TU27) from Prof. K. Sugamura and LAIR-2 (clone FMU-LAIR 2.1) from Dr. Boquan Jin. Conjugated mAb purchased were EGF-R-FITC (clone EGF-R1) from Insight Biotechnologyl; HLA-DR-FITC (clone L243), CD56-Alexa Fluor 488 (clone B159), CD3-FITC (clone SK7), CD14-PE (clone MφP9), CD132-PE (clone AG184) and LAIR-1-PE (clone DX26) all from Becton Dickinson; LILRB1-PE (clone HP-F1) from Beckman Coulter; LILRB3-PE (clone *293623*) from R&D Systems; CD45-PE-Cy5 (clone HI30) and IL-2Rβ-APC (clone TU27) both from BioLegend. Isotype controls used were the mAbs HIB19 and X40 (unconjugated and PE-conjugated) from Becton Dickinson, MOPC-21-APC from Biolegend and 20102-PE from R&D. Where indicated, binding of unlabelled mAbs was detected by polyclonal PE-conjugated secondary antibody to murine IgG (Sigma–Aldrich). Biotinylated polyclonal goat anti-LAIR-2 IgG was also obtained from R&D Systems.

### 2.3 Flow cytometry

Freshly isolated cells or those harvested by trypsin digestion of adherent trophoblast after overnight culture were resuspended in FACS buffer (1% FCS in PBS), incubated with human IgG (Sigma–Aldrich) followed by unlabelled primary monoclonal antibodies and then fluorochrome-conjugated polyclonal secondary antibodies. Free secondary antibody binding sites were blocked with murine immunoglobulin (Sigma–Aldrich) before staining with directly-conjugated mAb to identify leukocyte or trophoblast cell populations. Cells were analysed using FACScan or FACSCalibur flow cytometers with CellQuest software (Becton Dickinson), or sorted using a DakoCytomation MoFlo cytometer and Summit software.

### 2.4 Expression profiling by microarray

#### 2.4.1 RNA preparation

Total RNA was isolated from primary trophoblast or choriocarcinoma cell lines by lysis in Trizol (Invitrogen) followed by cleanup and DNAse treatment using RNeasy Micro kits (Qiagen). Biotinylated cRNA was synthesized using 100 ng of this total RNA using the Illumina RNA amplification kit (Ambion) according to the manufacturer’s instructions.

#### 2.4.2 Array hybridization

Labelled cRNA was hybridised to Illumina Human HT-12 V3 BeadArrays using the manufacturer’s standard protocol (*http://www.illumina.com/*). Illumina *BeadStudio* output, comprising background corrected and summarised expression scores for each microarray probe-set, was imported using functions of the *BeadArray* package for the *Bioconductor* (*http://bioconductor.org*) suite of software in the *R* statistical programming environment (*http://www.r-project.org*).

#### 2.4.3 Data processing

Signal intensities were converted to log2 expression units. Quantile normalisation [14], implemented in the *limma* package for Bioconductor, was employed to equalise summarised expression intensity distributions across all sample profiles. Probe-sets were annotated to gene targets using annotations from the *illuminaHumanv3.db* package for Bioconductor and the manufacturer’s own annotation package. Raw and processed data are available from the ArrayExpress microarray data repository (*http://www.ebi.ac.uk/arrayexpress/*) under accession number E-MTAB-429.

#### 2.4.4 Marker identification

Differential probe-set (or gene) expression between two sample groups was assessed via the output of a moderated *t*-test ([15], Bioconductor:*limma*). In order to reduce errors associated with multiple hypothesis testing on such a scale, the significance *p-values* obtained were converted to corrected *q-values* using the FDR method of Storey *et al* [16]. Probe-sets with associated *q* < 0.01 (FDR 1%) were deemed to exhibit significant differential expression between sample groups.

#### 2.4.5 Over-representation analysis

The statistical over-representation of Gene Ontology (GO; *http://www.geneontology.org*) Biological Process categories and Kyoto Encyclopoedia of Gene and Genomes pathways (KEGG; *http://www.genome.jp/kegg*) among genes deemed differentially expressed between sample group profiles was assessed via the hypergeometric statistics of the *GOStats* package for Bioconductor.

#### 2.4.6 Data Visualisation

Gene products within KEGG pathway maps were coloured according to up- or down-regulation of corresponding microarray probe-sets using the *Color Pathway* tool available via the KEGG website (*http://www.genome.jp/kegg/tool/color_pathway.html*).

### 2.5 Immunohistochemistry

Immunohistochemistry was performed as previously described [17]. Briefly, acetone-fixed frozen sections were blocked with serum of the species in which the secondary antibody was raised before staining with the antibodies described above. Binding was detected by biotinylated secondary antibodies followed by streptavidin-HRP (both Vector Labs) and developed with DAB substrate (Sigma–Aldrich). Slides were counterstained with Carazzi’s haematoxylin, cleared in xylene and mounted in DPX.

### 2.6 Sandwich enzyme-linked Immunosorbant assay (ELISA)

An ELISA for LAIR-2 was performed as previously described [18]. Briefly, urine samples or trophoblast culture supernatant were stored at −20 °C before analysis using the capture mAb FMU-LAIR 2.1 and detecting with purified biotinylated purified polyclonal antibody from R&D systems. Creatinine was measured using a commercial version of the Jaffe reaction (Assay Designs) to normalize for urine sample concentration. Despite changes in glomerular filtration rate, creatinine levels are constant throughout gestation in normal pregnancies [19] and we observed no differences in creatinine between urine from pregnant and non-pregnant women (not shown).

## 3 Results

### 3.1 Isolation of pure villous and extravillous trophoblast cells for analysis by microarray

The cells released from chorionic villi after a short trypsin digest are predominantly VT and these were purified immediately to >99% by flow sorting of EGFR+cells (Suppl Fig. 1). If villous cell isolates are instead cultured overnight on fibronectin, the adherent cells are mainly EVT and by flow sorting for HLA-G+cells, EVT of >99% purity were obtained (Suppl Fig. 1). Hofbauer cells (fetal macrophages) were positively excluded by labelling for CD14 or HLA-DR in both procedures, because they bind the mAbs used for trophoblast labelling via FcγR. Three separate preparations of VT and three of EVT were isolated from a total of six independent pregnancies. Choriocarcinoma cell lines JEG-3 and JAR cultured *in vitro* were also harvested on three different occasions. The twelve RNA samples isolated from these four cell types, were then hybridised independently to microarrays as described in the Materials and Methods.

### 3.2 Comparison of choriocarcinoma cell lines to primary trophoblast

**Fig. 1:**
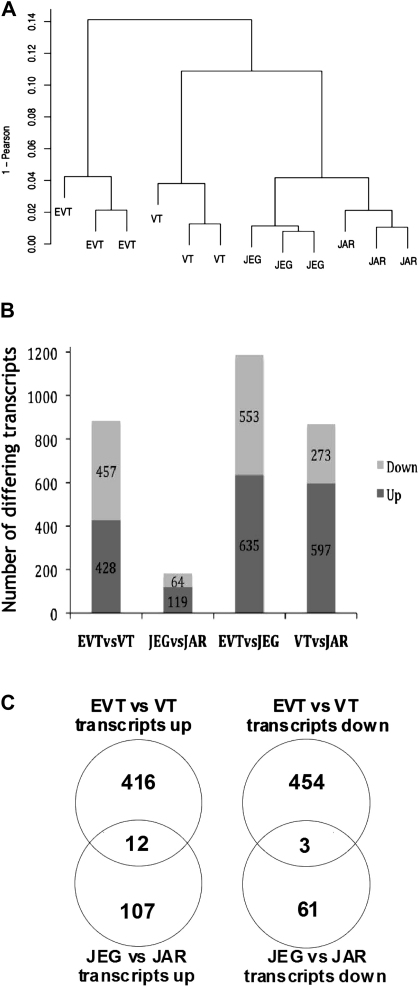
Comparison of gene expression profiles in primary EVT and VT with the choriocarcinoma cell lines JEG-3 and JAR. Unsupervised hierarchical clustering of the four cell types was performed using transcripts identified as showing variable expression across all samples (A). Organisation and length of the dendrogram branches reflect the degree of similarity between samples. (B). Identification of specific transcripts which differ significantly in expression (*q* < 0.01) by at least 4-fold up or down between cell types. 885 transcripts differ between EVT and VT by these criteria, compared with only 183 between JEG and JAR. 1188 transcripts differ between EVT and JEG and 870 between VT and JAR respectively. (C). The differences in expression between EVT and VT cells are not the same as the differences between JEG and JAR. For example, of the 457 transcripts at least 4-fold lower in EVT compared with VT, only 3 of these transcripts are also lower in JEG vs JAR.

JEG-3 expresses HLA-G, while JAR is HLA class I negative. Based on this phenotype, these choriocarcinoma cell lines are widely used as models of primary EVT and VT respectively. The expression profiles of primary EVT and VT cells were compared with the corresponding cell lines JEG-3 and JAR. Unsupervised hierarchical clustering produced a dendrogram in which each cell type formed individual groups, indicating a distinctive expression profile, but the two choriocarcinoma lines clustered together on a separate branch from either primary cell type (Fig. 1A). Over 850 transcripts differed significantly (*q* < 0.01) by at least 4-fold between EVT and VT, EVT and JEG-3, VT and JAR; whereas only 183 transcripts differed between JEG and JAR (Fig. 1B). Although HLA-G was up-regulated in both EVT and JEG-3 compared VT and JAR, the genes differentially expressed between the two lineages of primary trophoblast were not represented by those transcripts differing between the JEG-3 and JAR cell lines (Fig. 1C). Indeed, more transcripts differ between EVT and the ‘corresponding’ cell line JEG-3 than between EVT and VT. These choriocarcinoma cell lines appear, at the transcriptional level, to be poorly representative of the corresponding VT and EVT.

### 3.3 Comparison of EVT and VT

**Fig. 2:**
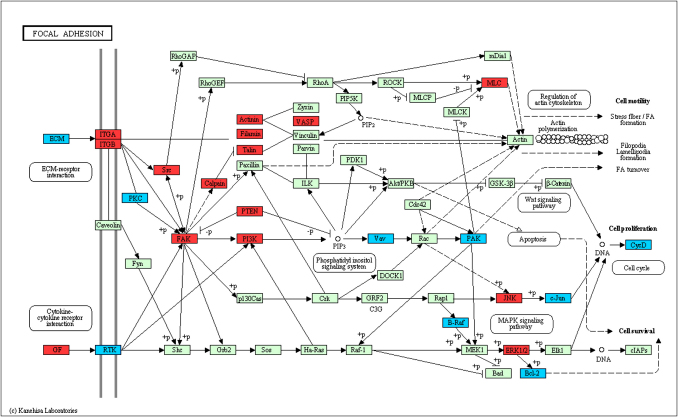
The Focal Adhesion Kinase (FAK) KEGG pathway has a significant number of transcripts up-regulated in EVT vs VT (*p* < 0.01). Individual transcripts significantly (*q* < 0.01) up-regulated in EVT are coloured red, transcripts that are signficantly lower in EVT compared to VT are coloured blue. Green indicates no significant change.

Comparison of the expression profiles of EVT and VT identified 3471 probes with *q* < 0.01 (FDR 1%) indicating differential expression. When classified into functional categories based on either GO annotation or KEGG pathways, over-representation analysis revealed that the most significantly up-regulated GO biological processes in EVT included ‘cell motion’ (63 transcripts), ‘immune response’ (71 transcripts) and ‘leukocyte adhesion’ (7 transcripts) (*p* < 0.0025, Supplementary Table 1). The most significantly down-regulated processes in EVT included metabolic processes such as ‘lipid metabolism’ and ‘fatty acid oxidation’ as well as ‘negative regulators of cell differentiation’. Specific functions represented by these transcripts were identified using the KEGG pathways which include known functional relationships between transcripts. Significantly up-regulated (*p* < 0.01) KEGG pathways included focal adhesion, extracellular matrix (ECM) receptor interactions, leukocyte transendothelial migration and cancer, whereas down-regulated pathways included amino acid metabolism, fatty acid metabolism and biosynthesis of steroids (Supplementary Table 2). These results show that as VT differentiate to EVT, there is co-ordinated up and down-regulation of specific pathways. The very significant up-regulation of more than 40 transcripts involved in the focal adhesion kinase (FAK) and ECM-receptor pathways, which are linked through the integrin receptors, clearly identifies a potentially important role for the focal adhesion kinase (PTK2) (Fig. 2). Several members of this pathway that are involved in cell proliferation are down-regulated on differentiation to EVT (*VAV*, *JUN* *and CYCD*). The FAK pathway also integrates transcripts involved in transducing signals that enhance cell migration (*PAK*, *MLC*, *ACTN4*), and these are up-regulated in EVT. Thus, this pathway offers novel insights into how FAK might be involved in the transition from proliferation in VT to post-mitotic, migratory EVT. Even in functions that have been well studied, such as migration, or ECM/receptor interactions we provide novel and more detailed information regarding the specific transcriptional changes underlying known differences in trophoblast function (Suppl Table 4).

### 3.4 Analysis of specific genes detected in EVT and VT

**Fig. 3:**
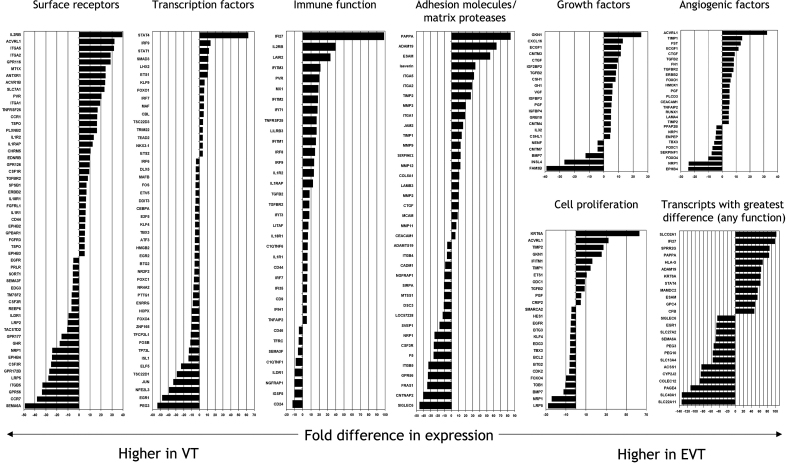
Genes differentially expressed between EVT and VT. A mean hybridization signal is used for each transcript, with all differences significant at *q* < 0.01. Only selected genes are shown here, for a complete list see Suppl Table 3. Genes were classified into functional categories based on Gene Ontology annotations.

Of the 3471 probes differing significantly between EVT and VT (*q* < 0.01), 885 transcripts differed by more than 4-fold (listed in Suppl Table 3). A selection of these transcripts, classified into functional catagories based on gene ontology annotations is presented in Fig. 3. The genes most strikingly up-regulated in VT were solute carrier family molecules, with *SLC40A1* (ferroportin-1) and *SLC22A11* > 130-fold and another 5 members of this family were expressed at levels more than 10-fold higher than in EVT. Also strongly up-regulated in VT were imprinted genes, *PEG3* and *PEG10* (>50-fold), the adhesion molecules integrin β5, siglec 6 and *FRAS1* (>30-fold), human endogenous retroviruses *HERV-FRD* and *ERVWE1*, the transcription factor *ELF5* and receptors for soluble factors such as colony stimulating factor 3, ephrin receptor, *EPHB4* and growth hormone receptor (all around 20-fold). Also strongly up-regulated in VT were poorly-characterised molecules such as palmitylated membrane protein 1 (*MPP1*) and contactin-associated protein-like 2 (*CNTNAP2*), both 40-fold higher in VT; lipoprotein receptor-related 5 (*LRP5*), the G-protein coupled receptors, *GPR56* and *GPR172B* (all around 30-fold) and the Ig-domain receptor, *ILDR1* (10-fold). Many of these represent transcripts not previously known to be highly expressed in VT.

The list of genes highly up-regulated in EVT compared to VT, contains many involved in immune function (Fig. 3). *HLA-G* along with the peptide loading chaperones *TAP1+2*, as well as *LAIR-2*, complement factor B (*CFB*) and the recently identified NK ligand *PVR* (CD155) are all up-regulated by at least 20-fold. Cytokine/chemokine receptors such as *CCR1*, *IL-1R2*, *IL-1RAP*, *IL-2RB*, *TNF-RSF25*, *ACVRL1* and *ACVR1B* are all increased, suggesting EVT acquires an enhanced ability to respond to the wide range of cytokines secreted in the decidua. Many cytokine-responsive factors are indeed increased including interferon-inducible proteins such as *IFITM2*, *IFIT1* and *IFI27* which is 100-fold higher in EVT. A second category of genes significantly up-regulated in EVT includes transcripts concerned with the regulation of cell migration: extracellular matrix proteases such as *PAPPA* (84-fold), *ADAM-19* (64-fold), laeverin (*AQPEP*) and *TIMP2* (both 30-fold) as well as *SERPINE2* which has the third highest expression level of all transcripts in EVT. Adhesion molecules that regulate cell migration are also up-regulated in EVT, including integrins α-1, -2 and -5 (∼25-fold), while integrins β-4 and β-5 are down-regulated as described previously [20]. We have also identified novel adhesion molecules not previously reported to be up-regulated on EVT: endothelial cell-specific adhesion molecule *ESAM* (55-fold higher in EVT) and vascular endothelial junction-associated molecule *JAM2* (16 fold). Both molecules are involved in regulating the adhesion of leukocytes to endothelium, and their up-regulation by EVT suggests trophoblast may utilize these molecules to interact with decidual leukocytes [21]. Different sets of solute carriers to those found in VT are up-regulated: *SLCO2A1* is over 100-fold higher in EVT and *SLC7A1*, *KCNK12* and *FXYD5* are all up 20-fold. These may reflect new transport requirements to support changes in metabolic and synthesis requirements between VT and EVT. Other genes were the transcription factor *STAT4*, the glypican G (both >50-fold) and the anthrax toxin receptor *ANTXR1* (25-fold). Also strongly up-regulated in EVT were less well characterised molecules such as small proline-rich protein 2G (*SPRR2G*), 85-fold higher in EVT; *MAMDC2* (55-fold), and the G-protein coupled receptor *GPR116* (>30-fold).

Two critical functions of EVT are their role in blood vessel remodeling and their interactions with maternal immune cells in the decidua. It is striking that transcripts encoding several novel growth factors previously unknown to be secreted by trophoblast are highly up-regulated in EVT. These included gastrokine 1 (*GKN1*), the neurosecretory protein VGF and CXCL16, whose receptor CXCR6 is also up-regulated in EVT by 3.7 fold. *TGFB2* and the corresponding receptors *TGFBR2* and *ACVRL1* were also highly up-regulated, again indicating co-ordinated regulation of this pathway leading to a potential autocrine loop in EVT. Several of these growth factors are potent regulators of angiogenesis. Connective tissue growth factor (CTGF) and placental growth factor (PGF) promote endothelial cell migration and proliferation, potently regulating angiogenesis and extracellular remodeling through their interactions with VEGF [22]. In contrast endothelial cell-specific growth factor (ECGF) promotes angiogenesis by a novel mechanism, in which the conversion of thymidine to thymine generates sugars such as 2-Deoxy-d-ribose which locally promote endothelial cell chemotaxis [23].

Genes highly expressed in both VT and EVT were also analysed. Transcripts encoding ribosomal, cellular organelle and cytoskeleton proteins as well as collagens and profilins were first excluded since these are abundant in all cells. A selection of other highly expressed genes identifying a profile characteristic of both VT and EVT is shown in Suppl Fig. 2. Transporters for calcium (*TPT1*), iron (*FTL*), glucose (*SLC2A1*), choline (*SLC44A4*) and other solutes are well represented (*S100P, KCNH6, DIRC2, CLDND1*). Several genes involved in amyloid processing appear: *ITM2B, BRI3, TMEM147*. Adrenomedullin (*ADM*), differentiation marker *MYADM*, the thrombin and putative progesterone receptors (*F2R* and *PGRMC2*) and imprinted genes (*H19* and *CD81*) are all detected at particularly high levels. Molecules with immune-related functions such as cytokine and chemokine receptors were prominent. A surprise finding was that the immunoglobulin and lectin family receptors *LAIR1, LILRB1, LILRB3* and *CLEC2D* were highly expressed in trophoblast, *LAIR1* being in the top 25 of all transcripts in both VT and EVT (Suppl Fig. 2).

### 3.5 Protein expression of LILRB1, LILRB3 and LAIR-1 is not detected on trophoblast cells

Leukocyte receptor complex genes *LILRB1, LILRB3* and *LAIR-1* are typically expressed on leukocytes and are all important in regulation of NK cell function [7]. We examined expression of these genes at the protein level, but no surface expression on trophoblast cells was observed by flow cytometry (Suppl Fig. 3A, B). Fetal Hofbauer cells contaminating the trophoblast cell preparations did stain for each of these leukocyte receptors, providing a clear positive control for these assays. In Suppl Fig. 3, the Hofbauer cells are EGF-R- and HLA-G-, their identification by triple labelling as CD45+ is not shown. Trophoblast also did not label for LAIR-1 in immuno-histological staining of a first trimester implantation site (Suppl Fig. 3C). The left panel shows an anchoring column of trophoblast derived from the villous placenta in cross section, with the distal trophoblast identified as EVT by HLA-G labelling. None of the trophoblast column labels for LAIR-1, ruling out intra-cellular protein expression.

### 3.6 Trophoblast up-regulate the IL-2 receptor β subunit on differentiation to an extravillous phenotype

**Fig. 4:**
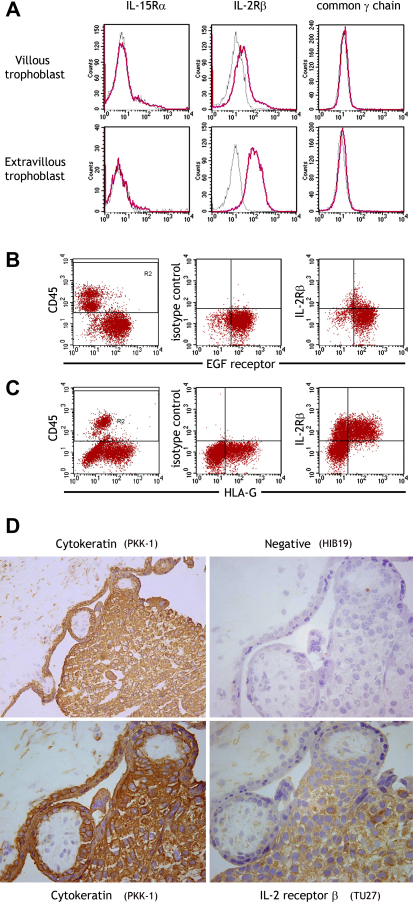
The IL-2 receptor β subunit is up-regulated on trophoblast cells as they differentiate from villous to extravillous phenotype. Upregulation of IL-2Rβ expression was validated by flow cytometry (A–C) and immunohistochemistry (D). Preparations of placental cells from normal first trimester pregnancies were gated on scatter, leukocytes excluded by CD45 labelling (gate R2) and subunits of the IL-15 receptor complex stained on villous (EGF-R+) or extravillous (HLA-G+) trophoblast cells (A). Dot plots are shown of IL-2Rβ subunit staining villous (B) and extravillous trophoblast (C). Shown here are cells from the same placenta which is representative of 3 pregnancies analysed. The IL-2Rβ was also localised histologically in sections of implantation site from the first trimester (D). The low power plan stained with cytokeratin shows villous mesenchyme (top left) and a column of EVT (bottom right) developing from placental villi (centre). Higher power pictures show strong IL-2Rβ staining detected on extravillous trophoblast migrating away from the villi, whereas staining was negligible on villous trophoblast and villous mesenchymal cells.

IL-2 receptor β was the most highly up-regulated surface receptor in EVT compared to VT (Fig. 3). We confirmed weak surface expression of IL2Rβ protein on VT but strong up-regulation on differentiation to EVT by flow cytometry of isolated cells and by histology *in vivo* (Fig. 4). IL2Rβ is a subunit in the receptor complex of IL-2 and IL-15, but only the latter has been found in the decidua [24]. The IL-15 receptor complex also includes IL-15Rα and common γ chain subunits but these were not detected on trophoblast (Fig. 4A). The IL-15Rα mAb did bind peripheral blood HLA-DR+ cells and the common γ chain mAb bound YT cells in control experiments (not shown).

### 3.7 Extravillous trophoblast secrete LAIR-2, soluble antagonist of the LAIR-1 receptor expressed by peripheral and decidual leukocytes

**Fig. 5:**
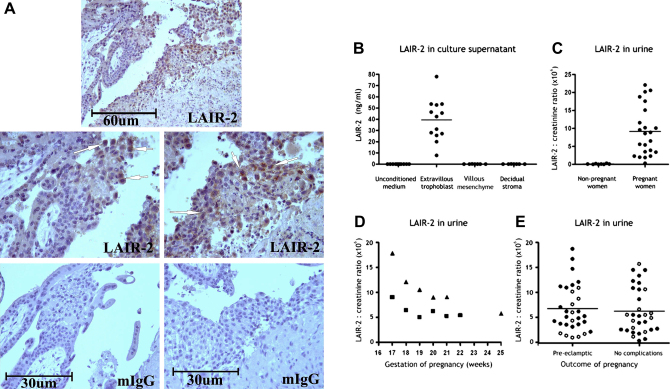
Extravillous trophoblast cells secrete LAIR-2. In histological staining of a first trimester implantation site extravillous trophoblast (arrowed) differentiating from a placental villus stain for LAIR-2 but not isotype control mAb (A). This secreted LAIR-2 can be detected by ELISA. LAIR-2 is detected in culture supernatant of extravillous trophoblast but not other placental or decidual cells from the implantation site of normal first trimester pregnancies (B). LAIR-2 in the urine of 23 pregnant women between 25 and 45 weeks gestation, but not non-pregnant women (C). Analysis of urine from two women throughout gestation shows LAIR-2 levels are highest before 20 weeks (D). No significant difference was found in urine LAIR-2 levels collected at 10–16 weeks gestation, from women who subsequently developed pre-eclampsia or who had pregnancies without complications (Mann–Whitney *U* test) (E). Filled circles are 14–16 week samples, open circles were collected at 10 weeks. There was no significant difference between samples from different timepoints, so the data was combined.

*LAIR-2*, a soluble antagonist of the LAIR-1 receptor was in the top 20 of all transcripts up-regulated in EVT compared to VT (33 fold higher, Suppl Table 3). EVT did label for LAIR-2 in immuno-histological staining of first trimester implantation site (Fig. 5A). By ELISA we detected LAIR-2 in the supernatants of isolated first trimester trophoblast cells (cultures were 70% HLA-G+), but not in supernatants of villous mesenchyme or decidual stromal cells from the same pregnancies (Fig. 5B). LAIR-2 protein is present in the urine of pregnant, but not non-pregnant women [18] and serial samples from two pregnant women showed that LAIR-2 levels in urine are highest early in gestation (Fig. 5C,D). LAIR-2 was the most down-regulated gene in a recent microarray study comparing first trimester chorionic villous samples from normal women with those who went on to develop pre-eclampsia [25]. We therefore compared LAIR-2 levels in the urine of gestationally-matched donors from healthy and pre-eclamptic pregnancies collected at between 10 and 16 weeks of gestation, but found no differences (Fig. 5E). Nonetheless, these data do strongly suggest that EVT secrete LAIR-2 as they invade into the decidua.

**Fig. 6:**
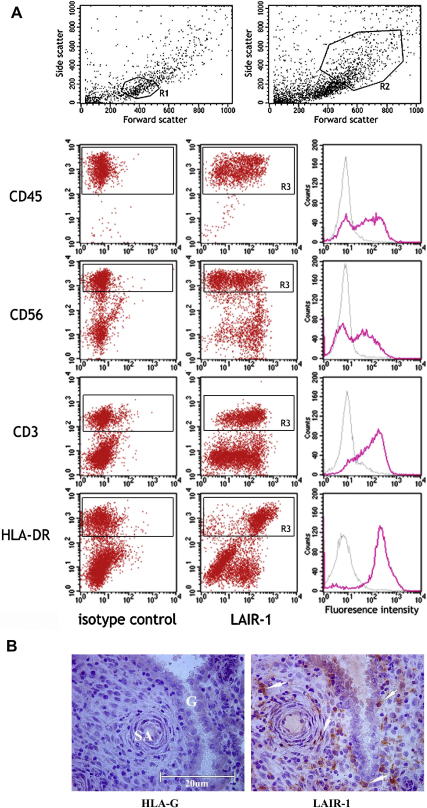
LAIR-1 expression in the decidua. Decidual leukocytes isolated from first trimester pregnancies were analysed by surface flow cytometry (A). The scatter gate R1 was used for CD45, CD56 and CD3 positive cells and R2 for HLA-DR+ myelomonocytic cells. Histograms show isotype control and LAIR-1 mAb staining to the leukocyte population discriminated (R3). Staining is representative of decidual leukocytes from 3 independent individuals. Histological staining of decidua adjacent to a first trimester implantation site is shown (B). No staining with an HLA-G mAb indicates the absence of extravillous trophoblast in this area. Immunoreactivity corresponding to LAIR-1 can be seen on maternal cells adjacent to arteries and glands or scattered throughout the decidua (right), consistent with LAIR-1 expression by maternal leukocytes. SA, spiral artery; G, uterine gland.

LAIR-2 has been shown to be a soluble antagonist of the closely related LAIR-1 receptor that is expressed by all peripheral leukocytes [26,27]. Using flow cytometry of first trimester decidual leukocytes, we found that LAIR-1 is expressed by all CD3+ and HLA-DR+ cells, and ∼50% of the CD56 + cell population (Fig. 6A). Immuno-histological staining of the decidua basalis showed that these LAIR-1+ leukocytes are present underlying glandular epithelium and around blood vessels as well as scattered throughout the decidua (Fig. 6B).

## 4 Discussion

Our transcriptional analysis of villous and extravillous trophoblast cells isolated directly from the first trimester implantation site has reassuringly identified many genes previously known to be differentially expressed in these two cell types. Genes more highly expressed in EVT are *CD9* [28] and *CD155* [8], pregnancy-associated plasma protein A [29], matrix metallopeptidases such as *MMP-9*, laeverin and *ADAM19* as well as their tissue inhibitor *TIMP-2* [30–32]. Transcripts increased in VT include *CD46* [33], *PEG3* [34], placental collectin 12 [35], *PLAC8* [36], ferroportin-1 (*SLC40A1*) [37], folate receptor 1 *(FOLR1*) [38], *SIGLEC6* [39] and the transcription factor *ELF5* [40]. Further genes such as *CD274* [41], glucose transporter *GLUT-1* [42], calcium uptake molecules *TPT1* [43] and *S100P* [44], and the thrombin receptor *F2R* [45] were detected in both trophoblast cell types as has been reported before. Agreement with these earlier findings gives confidence that the many new genes we found expressed by trophoblast sub-populations will provide valid and interesting leads for future functional studies.

A similar recent microarray study which sought to compare VT and EVT identified the heme-oxygenase 1 pathway as down-regulated in invasive EVT [46]. Apart from a more comprehensive probe set, a major difference between this study and our own is that we isolated trophoblast cells with minimal *in vitro* culture. VT was used immediately and EVT after only 12 h culture. This compares with 1 day (VT) and 3 days (EVT) of culture in the previous study. Our aim was to reduce to a minimum the transcriptional changes occurring *ex vivo*. In the previous study Bilban et al obtained VT by HLA class I negative selection with magnetic beads and EVT by manual picking of outgrowths from 3-day cultures of villous explants. The purity of our flow-sorted trophoblast populations is higher (>99%) and these methodological differences as well as the extent of differentiation into EVT may explain why our results are different. Although we replicate many of the findings by Bilban et al, the magnitude of the differences between VT and EVT tend to be much higher in the present study. *HLA-G, ADAM-19* and *PAPPA* are more than 60-fold higher in EVT compared to VT, but only 3-fold in the earlier study. The analysis presented here also identifies 3433 transcripts showing at least a two-fold difference between EVT and VT, compared to 991 in the previous study.

JEG-3 and JAR are choriocarcinoma cell lines widely used as models of primary EVT and VT respectively, but the expression profiles of these choriocarcinoma cell lines very poorly represented the primary trophoblast. Syncytin (*ERVWE1*) was one of only three transcripts up-regulated in both JAR and VT, compared to JEG-3 and EVT, indicating JAR may be of use to study the role of this endogenous retroviral molecule in fusion of the syncytial villous trophoblast layer [47]. *HLA-G* was up-regulated in JEG-3 and EVT and this line has already been recognized as useful for study of EVT HLA class I molecules [48]. The choriocarcinoma lines are therefore of use to study some limited aspects of trophoblast immunobiology, but transformation *in vivo* and their subsequent culture have resulted in transcriptional profiles that are poorly representative of primary trophoblast. Indeed JEG-3 was derived from the cerebral metastasis of a choriocarcinoma in 1959, and has subsequently been cultured for hundreds of passages in hamster cheek pouches and laboratory tissue culture. It may be possible to establish culture conditions in which the cell lines represent primary trophoblast more closely, but these microarray profiles suggest any phenotypic or functional analysis performed on the lines must be validated in primary cells. For example, although JEG-3 does express the non-classical HLA-G molecule, its conformation on the surface of JEG-3 differs from that on primary EVT in a manner that has functional consequences [49]. A recent analysis of choriocarcinoma and placental cell lines transformed *in vitro* supports this conclusion [57].

Comparison of the expression profiles of EVT and VT identified 3471 probes with significantly different expression. To help understand the biological processes they represented, these transcripts were classified into functional categories based on either GO annotation or KEGG pathways. Although functions such as cell motion and immune response were previously known in EVT, this revealed a much more complete picture of the transcripts involved. An example is FAK, which has previously been studied in trophoblast invasion [50], but the significant up-regulation of more than 40 transcripts involved in the focal adhesion and ECM-receptor pathways gives a more complete understanding. This new global analysis shows that many pathways are co-ordinately regulated. It also identifies many new transcripts in these pathways where changes in expression may alter trophoblast function. The co-ordinated changes in pathways lead us to examine what transcription factors altered during the transition from VT to EVT. VT is characterised by high expression of six transcription factors expressed at 20–55 fold higher than in EVT. Differentiation to EVT is associated with down-regulation of these, and up-regulation of *STAT4*, *IRF9* and *STAT1* (>10 fold). We speculate that changes in transcription factors such as STAT4 may serve to co-ordinate many of the other gene expression changes that we document. In support of this, the promoters of many of the genes most highly up-regulated in EVT are predicted to contain STAT4 response elements. These include *STAT1*, *ETS1*, *CTGF*, and *ESAM*, suggesting STAT4 may act as a ‘master regulator’ of EVT differentiation.

Some of these genes identified might also serve as new markers of trophoblast subsets. The microarray hybridization signal for EGF-R, used here and in other studies to isolate VT, is only 5 fold higher in VT than EVT. In contrast, the surface protein semaphorin 6A is more than 50-fold higher in VT than EVT, with *SEMA6A* transcript levels 10 times higher in VT than *EGF-R*. We also found high levels of expression in both VT and EVT of specific IgSF genes from the leukocyte receptor complex (LRC): *LILRB1, LILRB3* and *LAIR-1*. These molecules are usually expressed by leukocytes and indeed no protein expression was detected by flow cytometry or immunohistochemistry, despite high transcript levels they appear not to be translated in trophoblast. In contrast, increased *IL-2Rβ* transcripts in EVT were confirmed at the protein level by flow cytometry and immunohistology. There was no evidence of expression of the common γ chain or IL-15Rα, raising the question of whether the IL-2 receptor β subunit alone could be functional. When transfected alone, IL-2Rβ forms a homodimer able to bind IL-2 [51]. Complexes of IL-2Rβ, formed by the intra-cellular domain of IL-2Rβ fused to the extracellular domain of c-kit, have been shown to activate the Jak2 pathway [52] whereas IL-2 receptor signalling through the conventional α/β/γ complex leads to phosphorylation of Jak1 and 3. Similar experiments have not been performed with IL-15, which is synthesized by decidual cells and thus more likely than IL-2 to stimulate the trophoblast IL-2Rβ *in vivo*. Alternatively, IL-2Rβ may be involved in signalling through an as yet undiscovered receptor complex.

An important aim of this study was to identify novel ways in which EVT might interact with decidual leukocytes. IL-32 mRNA was up-regulated in EVT and is known to be secreted by epithelial cells following stimulation by IFN-γ. IL-32 can stimulate its own release from NK cells in a feedback loop and potently induces macrophages to secrete TNF-α and IL-8 [53]. This suggests EVT can regulate the function of uNK cells and macrophages by secretion of cytokines. NK cell function is also controlled by activating and inhibitory receptors, which recognise MHC and non-MHC ligands in an immunological synapse when NK cells directly contact target cells [7]. Extravillous trophoblast is known to express HLA-G, HLA-C, HLA-E and non-MHC ligands such as E-cadherin, PVR and CD112 [2,8]. We also demonstrate here high expression in both VT and EVT of transcripts of *CLEC2D* (LLT-1 or OCIL), ligand for the activating NK cell receptor CD161 (NKR-P1A). *CLEC1A*, an orphan receptor from the same family was also highly expressed. Although these results require validation at the protein level, they reveal new potential ligand/receptor interactions between EVT and NK cells during placentation.

Another mechanism by which EVT may regulate the function of decidual leukocytes is the apparent secretion of LAIR-2 by EVT. Protein expression of LAIR-2 by EVT was confirmed by histological staining of anchoring cytotrophoblast columns, in agreement with a recent study [54]. ELISA detected secreted LAIR-2 in trophoblast culture supernatants and urine of pregnant women. *LAIR-2* is encoded adjacent to *LAIR-1* in the LRC; LAIR-1 is an inhibitory receptor binding to extracellular matrix collagen [27]. LAIR-2 is a secreted protein with a single Ig-domain highly homologous to that of LAIR-1, and it antagonises LAIR-1 binding to collagen [18]. LAIR-1 is expressed by almost all peripheral leukocytes [26] and we show that it is expressed on at least 50% of decidual leukocytes. Leukocytes in the systemic circulation are usually not exposed to collagen but as they migrate from the blood, LAIR-1 binding to collagen may deliver an inhibitory signal necessary in the decidual microenvironment [27]. LAIR-2 secreted by invading EVT may increase decidual leukocyte responsiveness by blocking LAIR-1 binding specifically at the maternal–fetal interface, where maternal leukocytes contact allogeneic fetal EVT. Alternatively recent findings suggest LAIR-2 may have a role in local inhibition of platelet activation and aggregation [56]. A potential role of LAIR-2 in trophoblast invasion is clearly suggested by a microarray study comparing gene expression in chorionic villous samples of normal first trimester pregnancies with those that subsequently developed pre-eclampsia [25], a disease caused by inadequate trophoblast invasion [55]. *LAIR-2* was the most down-regulated gene in placentas from women that went on to develop pre-eclampsia later in pregnancy. We failed to find any differences in urinary LAIR-2 between normal and pre-eclamptic pregnancies from samples taken early in pregnancy. These results suggest a novel mechanism by which EVT may regulate the function of leukocytes locally in the decidua, as well as the wider maternal immune system. Further work is clearly warranted to characterise the functions of LAIR-1 and trophoblast LAIR-2 binding to extracellular matrix collagens at the implantation site.

In summary, we provide a detailed analysis of the transcriptional changes associated with the differentiation of villous to extravillous trophoblast. Using highly purified primary trophoblast cells, we identified over 3000 transcripts that are differentially expressed between these trophoblast subsets. Many of the new genes that are altered identify novel pathways that change, or add new players to previously studied functions such as migration or immune modulation. The differentiation from VT to EVT is characterised by alterations in transcription factors, with STAT4 up-regulation identified as a likely key regulator orchestrating these changes. We show that EVT secretes LAIR-2, a molecule that can regulate the functions of the inhibitory receptor LAIR-1 expressed on decidual T cells, macrophages and NK cells. As LAIR-2 is also detectable in urine from pregnant women, trophoblast-derived LAIR-2 may also have wider systemic effects during early pregnancy.

## Appendix Supplementary data

Suppl Fig. 1: Flow sorting of villous and extravillous trophoblast cells for analysis by microarray. Preparations of placental cells from normal first trimester pregnancies were gated on scatter and villous (EGFR+ CD14-) or extravillous (HLA-G+ HLA-DR-) trophoblast cells sorted to >99% purity. VT were isolated immediately, EVT were sorted following overnight culture of VT to permit differentiation to EVT. Boxed subsets indicate cells that were sorted. Sorted cell populations were reanalysed after the sort to confirm their purity.

Suppl Fig. 2: Transcripts highly expressed in both EVT and VT. Mean hybridization signal is shown for each transcript in VT and EVT samples. The background signal was approximately 6.1. All hybridization signals are given log 2. Transcripts encoding ribosomal, cellular organelle and structural proteins abundant in all cells are not shown.

Suppl Fig. 3: Protein expression of the leukocyte receptor complex genes LILRB1, LILRB3 and LAIR-1 is not detected in trophoblast cells. Preparations of placental cells from normal first trimester pregnancies were gated on scatter by flow cytometry and trophoblast cells identified by labelling EGF-R on villous (A) or HLA-G on extravillous trophoblast (B). Neither trophoblast population stained for the leukocyte receptor complex genes LILRB1, LILRB3 or LAIR-1. Histograms show isotype control and leukocyte receptor mAb staining to the trophoblast population identified. Villous and extravillous trophoblast from the same placenta are shown here, which are representative of 3 pregnancies analysed. Histological staining of a first trimester implantation site with an ×40 objective (C). Left panel stained for HLA-G, showing arrowed extravillous trophoblast differentiating from a placental villous. A serial section stained for LAIR-1 in the right hand panel shows that all trophoblast are negative. VM, villous mesencyme.

Suppl Table 1 : GO biological processes over-represented in differences between the transcriptional profile of EVT and VT, with a significance threshold of *p* < 0.0025.

Suppl Table 2 : KEGG pathways over-represented in differences between the transcriptional profile of EVT and VT, with a significance threshold of *p* < 0.01.

Suppl Table 3 : Transcripts different between EVT and VT. 885 transcripts were significantly different at *q* < 0.01 and differing by >4 fold. Individual hybridization signals are shown for each sample, following normalization (log2 values) and the magnitude of the fold change (EVT vs VT) is then given.

Suppl Table 4 : Co-ordinated regulation of genes in KEGG pathways. Average hybridization signals are shown for transcripts significantly altered in Fatty-acid metabolism (KEGG 00071), ECM-receptor interactions (KEGG 4512), Focal Adhesion (KEGG 4510) and Fatty-acid metabolism KEGG 00071) pathways.

## References

[1] Moffett A., Loke C. (2006 Aug). Immunology of placentation in eutherian mammals. Nat Rev Immunol.

[2] Moffett-King A. (2002 Sep). Natural killer cells and pregnancy. Nat Rev Immunol.

[3] Genbacev O., Zhou Y., Ludlow J.W., Fisher S.J. (1997 Sep 12). Regulation of human placental development by oxygen tension. Science.

[4] Hanna J., Goldman-Wohl D., Hamani Y., Avraham I., Greenfield C., Natanson-Yaron S. (2006 Sep). Decidual NK cells regulate key developmental processes at the human fetal-maternal interface. Nat Med.

[5] Hiby S.E., Walker J.J., O’shaughnessy K.M., Redman C.W., Carrington M., Trowsdale J. (2004 Oct 18). Combinations of maternal KIR and fetal HLA-C genes influence the risk of preeclampsia and reproductive success. J Exp Med.

[6] Hiby SE, Apps R, Sharkey AM, Farrlee LE, Gardner L, Mulder A, et al, Moffett A.Maternal activating KIR protect against human reproductive failure mediated by fetal HLA-C2. J Clin Invest 2010 Oct 25 [Epub ahead of print]. 10.1172/JCI43998 PMC2964995 20972337

[7] Vivier E., Tomasello E., Baratin M., Walzer T., Ugolini S. (2008 May). Functions of natural killer cells. Nat Immunol.

[8] Vacca P., Cantoni C., Prato C., Fulcheri E., Moretta A., Moretta L. (2008 Nov). Regulatory role of NKp44, NKp46, DNAM-1 and NKG2D receptors in the interaction between NK cells and trophoblast cells. Evidence for divergent functional profiles of decidual versus peripheral NK cells. Int Immunol.

[9] Male V., Trundley A., Gardner L., Northfield J., Chang C., Apps R. (2010). Natural killer cells in human pregnancy. Methods Mol Biol.

[10] Jokhi P.P., King A., Loke Y.W. (1994 Jul). Reciprocal expression of epidermal growth factor receptor (EGF-R) and c-erbB2 by non-invasive and invasive human trophoblast populations. Cytokine.

[11] Apps R., Gardner L., Moffett A. (2008 Jul). A critical look at HLA-G. Trends Immunol.

[12] Loke Y.W., King A., Burrows T., Gardner L., Bowen M., Hiby S. (1997 Aug). Evaluation of trophoblast HLA-G antigen with a specific monoclonal antibody. Tissue Antigens.

[13] Menier C., Saez B., Horejsi V., Martinozzi S., Krawice-Radanne I., Bruel S. (2003 Mar). Characterization of monoclonal antibodies recognizing HLA-G or HLA-E: new tools to analyze the expression of nonclassical HLA class I molecules. Hum Immunol.

[14] Irizarry R.A., Bolstad B.M., Collin F., Cope L.M., Hobbs B., Speed T.P. (2003 Feb 15). Summaries of Affymetrix GeneChip probe level data. Nucleic Acids Res.

[15] Smyth G.K. (2004). Linear models and empirical bayes methods for assessing differential expression in microarray experiments. Stat Appl Genet Mol Biol.

[16] Storey J.D., Tibshirani R. (2003 Aug 5). Statistical significance for genomewide studies. Proc Natl Acad Sci U S A.

[17] Jokhi P.P., Chumbley G., King A., Gardner L., Loke Y.W. (1993). Expression of the colony stimulating factor-1 receptor (c-fms product) by cells at the human uteroplacental interface. Lab Invest.

[18] Lebbink R.J., van den Berg M.C., de Ruiter T., Raynal N., van Roon J.A., Lenting P.J. (2008 Feb 1). The soluble leukocyte-associated Ig-like receptor (LAIR)-2 antagonizes the collagen/LAIR-1 inhibitory immune interaction. J Immunol.

[19] Waugh J., Bell S.C., Kilby M.D., Lambert P.C., Blackwell C.N., Shennan A. (2003 Feb). Urinary microalbumin/creatinine ratios: reference range in uncomplicated pregnancy. Clin Sci (Lond).

[20] Damsky C.H., Librach C., Lim K.H., Fitzgerald M.L., McMaster M.T., Janatpour M. (1994 Dec). Integrin switching regulates normal trophoblast invasion. Development.

[21] Wegmann F., Petri B., Khandoga A.G., Moser C., Khandoga A., Volkery S. (2006 Jul 10). ESAM supports neutrophil extravasation, activation of Rho, and VEGF-induced vascular permeability. J Exp Med.

[22] Inoki Isao, Shiomi Takayuki, Hashimoto Gakuji, Enomoto Hiroyuki, Nakamura Hiroyuki, Makino Ken-ichi (2002). Connective tissue growth factor binds vascular endothelial growth factor (VEGF) and inhibits VEGF-induced angiogenesis. FASEB J (United States).

[23] Nakajima Y., Madhyastha R., Maruyama M. (2009 Feb). 2-Deoxy-D-ribose, a downstream mediator of thymidine phosphorylase, regulates tumor angiogenesis and progression. Anticancer Agents Med Chem.

[24] Jokhi P.P., King A., Loke Y.W. (1997 Feb). Cytokine production and cytokine receptor expression by cells of the human first trimester placental-uterine interface. Cytokine.

[25] Founds S.A., Conley Y.P., Lyons-Weiler J.F., Jeyabalan A., Hogge W.A., Conrad K.P. (2009 Jan). Altered global gene expression in first trimester placentas of women destined to develop preeclampsia. Placenta.

[26] Meyaard L., Adema G.J., Chang C., Woollatt E., Sutherland G.R., Lanier L.L. (1997 Aug). LAIR-1, a novel inhibitory receptor expressed on human mononuclear leukocytes. Immunity.

[27] Lebbink R.J., de Ruiter T., Adelmeijer J., Brenkman A.B., van Helvoort J.M., Koch M. (2006 Jun 12). Collagens are functional, high affinity ligands for the inhibitory immune receptor LAIR-1. J Exp Med.

[28] Hirano T., Higuchi T., Ueda M., Inoue T., Kataoka N., Maeda M. (1999 Feb). CD9 is expressed in extravillous trophoblasts in association with integrin alpha3 and integrin alpha5. Mol Hum Reprod.

[29] Sun I.Y., Overgaard M.T., Oxvig C., Giudice L.C. (2002 Nov). Pregnancy-associated plasma protein A proteolytic activity is associated with the human placental trophoblast cell membrane. J Clin Endocrinol Metab.

[30] Okamoto T., Niu R., Yamada S., Osawa M. (2002 Apr). Reduced expression of tissue inhibitor of metalloproteinase (TIMP)-2 in gestational trophoblastic diseases. Mol Hum Reprod.

[31] Fujiwara H., Higuchi T., Yamada S., Hirano T., Sato Y., Nishioka Y. (2004 Jan 23). Human extravillous trophoblasts express laeverin, a novel protein that belongs to membrane-bound gluzincin metallopeptidases. Biochem Biophys Res Commun.

[32] Zhao M., Qiu W., Li Y., Sang Q.A., Wang Y. (2009 Aug). Dynamic change of Adamalysin 19 (ADAM19) in human placentas and its effects on cell invasion and adhesion in human trophoblastic cells. Sci China C Life Sci.

[33] Purcell D.F., McKenzie I.F., Lublin D.M., Johnson P.M., Atkinson J.P., Oglesby T.J. (1990 Jun). The human cell-surface glycoproteins HuLy-m5, membrane co-factor protein (MCP) of the complement system, and trophoblast leucocyte-common (TLX) antigen, are CD46. Immunology.

[34] Hiby S.E., Lough M., Keverne E.B., Surani M.A., Loke Y.W., King A. (2001 May 1). Paternal monoallelic expression of PEG3 in the human placenta. Hum Mol Genet.

[35] Selman L., Skjodt K., Nielsen O., Floridon C., Holmskov U., Hansen S. (2008 Jun). Expression and tissue localization of collectin placenta 1 (CL-P1, SRCL) in human tissues. Mol Immunol.

[36] Galaviz-Hernandez C., Stagg C., de Ridder G., Tanaka T.S., Ko M.S., Schlessinger D. (2003 May 8). Plac8 and Plac9, novel placental-enriched genes identified through microarray analysis. Gene.

[37] Bastin J., Drakesmith H., Rees M., Sargent I., Townsend A. (2006 Sep). Localisation of proteins of iron metabolism in the human placenta and liver. Br J Haematol.

[38] Solanky N., Requena Jimenez A., D’Souza S.W., Sibley C.P., Glazier J.D. (2010 Feb). Expression of folate transporters in human placenta and implications for homocysteine metabolism. Placenta.

[39] Brinkman-Van der Linden E.C., Hurtado-Ziola N., Hayakawa T., Wiggleton L., Benirschke K., Varki A. (2007 Sep). Human-specific expression of Siglec-6 in the placenta. Glycobiology.

[40] Hemberger M., Udayashankar R., Tesar P., Moore H., Burton G.J. (2010 Jun 15). ELF5-enforced transcriptional networks define an epigenetically regulated trophoblast stem cell compartment in the human placenta. Hum Mol Genet.

[41] Petroff M.G., Chen L., Phillips T.A., Hunt J.S. (2002 Apr). B7 family molecules: novel immunomodulators at the maternal-fetal interface. Placenta.

[42] Ericsson A., Hamark B., Powell T.L., Jansson T. (2005 Feb). Glucose transporter isoform 4 is expressed in the syncytiotrophoblast of first trimester human placenta. Hum Reprod.

[43] Arcuri F., Papa S., Meini A., Carducci A., Romagnoli R., Bianchi L. (2005 Oct). The translationally controlled tumor protein is a novel calcium binding protein of the human placenta and regulates calcium handling in trophoblast cells. Biol Reprod.

[44] Parkkila S., Pan P.W., Ward A., Gibadulinova A., Oveckova I., Pastorekova S. (2008 Feb 18). The calcium-binding protein S100P in normal and malignant human tissues. BMC Clin Pathol.

[45] Even-Ram S.C., Grisaru-Granovsky S., Pruss D., Maoz M., Salah Z., Yong-Jun Y. (2003 May). The pattern of expression of protease-activated receptors (PARs) during early trophoblast development. J Pathol.

[46] Bilban M., Haslinger P., Prast J., Klinglmüller F., Woelfel T., Haider S. (2009 Feb). Identification of novel trophoblast invasion-related genes: heme oxygenase-1 controls motility via peroxisome proliferator-activated receptor gamma. Endocrinology.

[47] Mi S., Lee X., Li X., Veldman G.M., Finnerty H., Racie L. (2000 Feb 17). Syncytin is a captive retroviral envelope protein involved in human placental morphogenesis. Nature.

[48] Apps R., Murphy S.P., Fernando R., Gardner L., Ahad T., Moffett A. (2009 May). Human leucocyte antigen (HLA) expression of primary trophoblast cells and placental cell lines, determined using single antigen beads to characterize allotype specificities of anti-HLA antibodies. Immunology.

[49] Apps R., Gardner L., Sharkey A.M., Holmes N., Moffett A. (2007 Jul). A homodimeric complex of HLA-G on normal trophoblast cells modulates antigen-presenting cells via LILRB1. Eur J Immunol.

[50] Ilić D., Genbacev O., Jin F., Caceres E., Almeida E.A., Bellingard-Dubouchaud V. (2001 Jul). Plasma membrane-associated pY397FAK is a marker of cytotrophoblast invasion in vivo and in vitro. Am J Pathol.

[51] Pillet A.H., Juffroy O., Mazard-Pasquier V., Moreau J.L., Gesbert F., Chastagner P. (2008 Mar). Human IL-Rbeta chains form IL-2 binding homodimers. Eur Cytokine Netw.

[52] Ferrag F., Pezet A., Chiarenza A., Buteau H., Nelson B.H., Goffin V. (1998 Jan 2). Homodimerization of IL-2 receptor beta chain is necessary and sufficient to activate Jak2 and downstream signaling pathways. FEBS Lett.

[53] Conti P., Youinou P., Theoharides T.C. (2007 Oct). Modulation of autoimmunity by the latest interleukins (with special emphasis on IL-32). Autoimmun Rev.

[54] Founds S.A., Fallert-Junecko B., Reinhart T.A., Conley Y.P., Parks W.T. (2010 Oct). LAIR2 localizes specifically to sites of extravillous trophoblast invasion. Placenta.

[55] Khong T.Y., De Wolf F., Robertson W.B., Brosens I. (1986). Inadequate maternal vascular response to placentation in pregnancies complicated by pre-eclampsia and by small-for-gestational age infants. Br J Obstet Gynaecol.

[56] Lenting P.J., Westerlaken G.H., Denis C.V., Akkerman J.W., Meyaard L. (2010 Aug 13). Efficient inhibition of collagen-induced platelet activation and adhesion by LAIR-2, a soluble Ig-like receptor family member. PLoS One.

[57] Bilban M., Tauber S., Haslinger P., Pollheimer J., Saleh L., Pehamberger H. (2010 Nov). Trophoblast invasion: assessment of cellular models using gene expression signatures. Placenta.

