# Supplementary figures and images for: Genome-wide expression profile of first trimester villous and extravillous human trophoblast cells
Source: Placenta. 2011 Jan;32(1-3):33–43. doi: 10.1016/j.placenta.2010.10.010 (PMC3065343; doi:10.1016/j.placenta.2010.10.010)

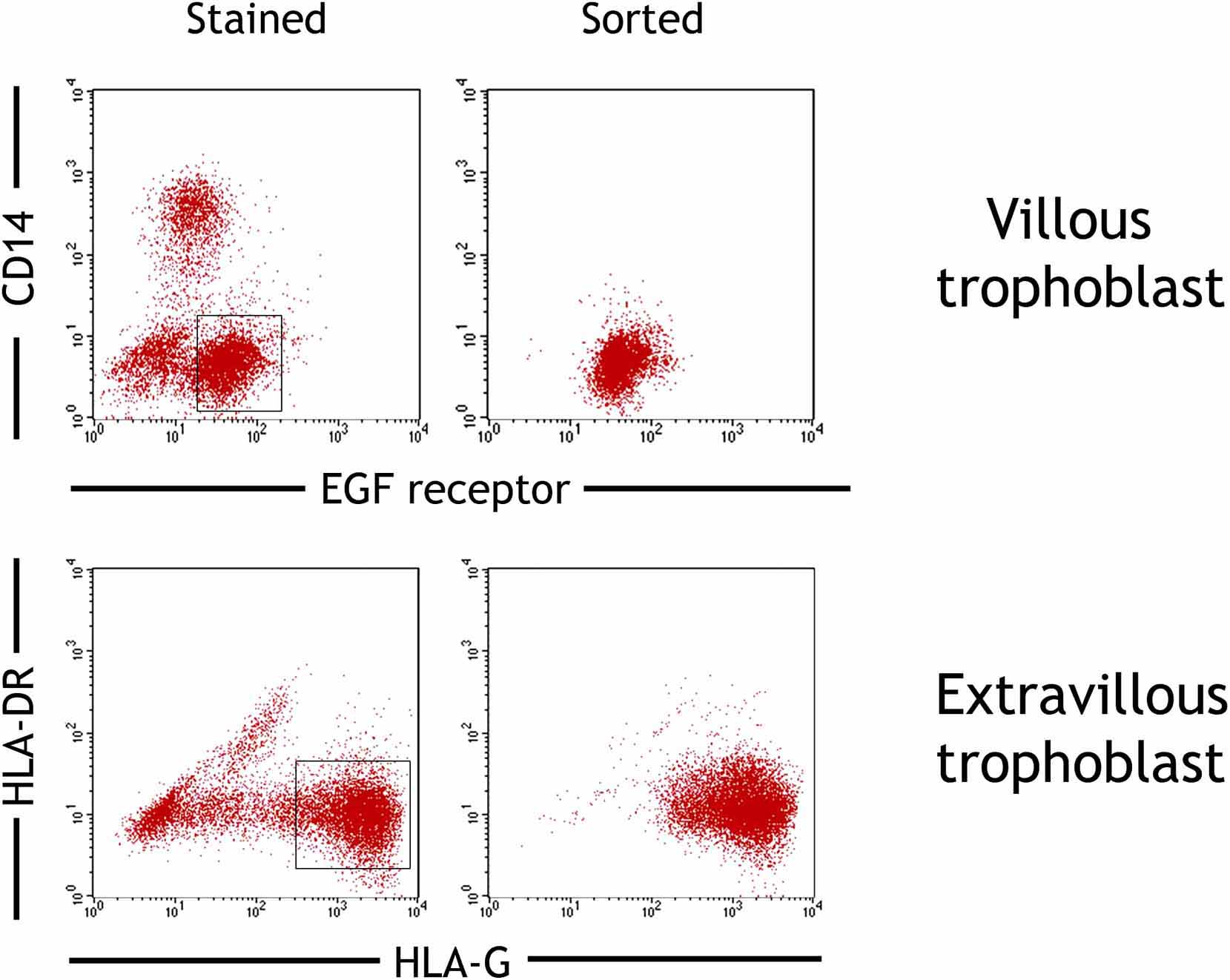

Supplement: Supplementary file 1 — Suppl Fig. 1: Flow sorting of villous and extravillous trophoblast cells for analysis by microarray. Preparations of placental cells from normal first trimester pregnancies were gated on scatter and villous (EGFR+ CD14-) or extravillous (HLA-G+ HLA-DR-) trophoblast cells sorted to >99% purity. VT were isolated immediately, EVT were sorted following overnight culture of VT to permit differentiation to EVT. Boxed subsets indicate cells that were sorted. Sorted cell populations were reanalysed after the sort to confirm their purity. [file figs1.jpg]

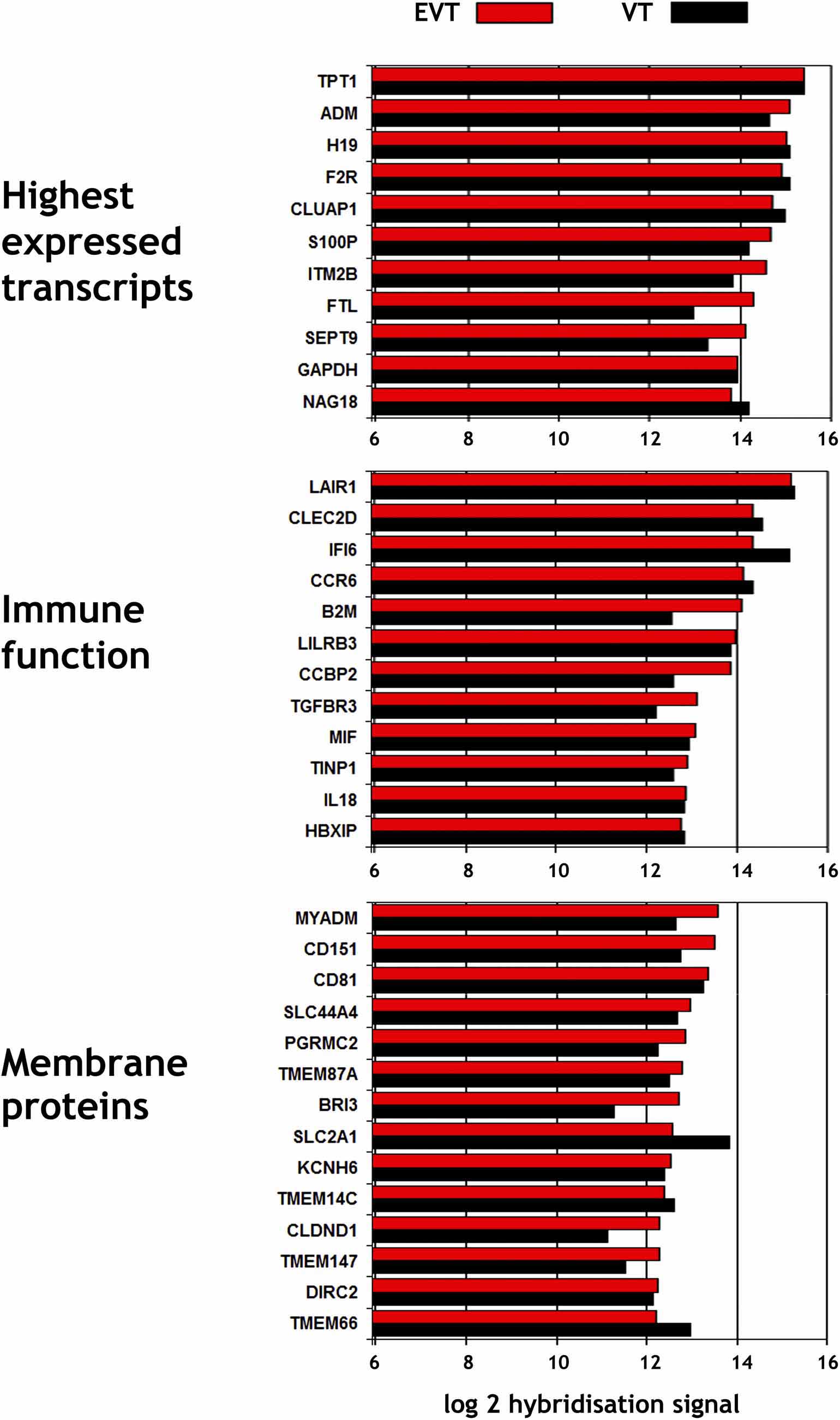

Supplement: Supplementary file 2 — Suppl Fig. 2: Transcripts highly expressed in both EVT and VT. Mean hybridization signal is shown for each transcript in VT and EVT samples. The background signal was approximately 6.1. All hybridization signals are given log 2. Transcripts encoding ribosomal, cellular organelle and structural proteins abundant in all cells are not shown. [file figs2.jpg]

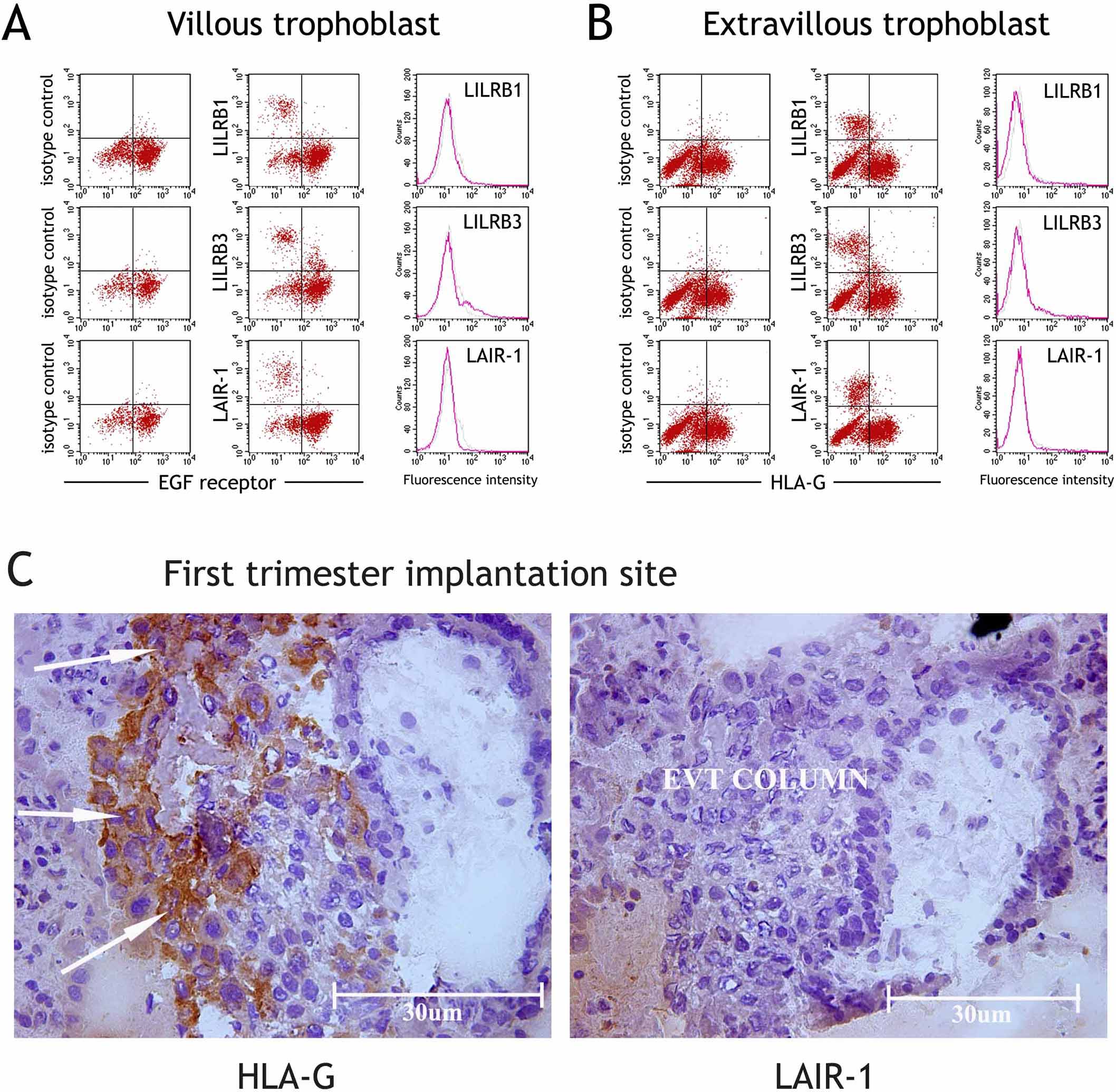

Supplement: Supplementary file 3 — Suppl Fig. 3: Protein expression of the leukocyte receptor complex genes LILRB1, LILRB3 and LAIR-1 is not detected in trophoblast cells. Preparations of placental cells from normal first trimester pregnancies were gated on scatter by flow cytometry and trophoblast cells identified by labelling EGF-R on villous (A) or HLA-G on extravillous trophoblast (B). Neither trophoblast population stained for the leukocyte receptor complex genes LILRB1, LILRB3 or LAIR-1. Histograms show isotype control and leukocyte receptor mAb staining to the trophoblast population identified. Villous and extravillous trophoblast from the same placenta are shown here, which are representative of 3 pregnancies analysed. Histological staining of a first trimester implantation site with an ×40 objective (C). Left panel stained for HLA-G, showing arrowed extravillous trophoblast differentiating from a placental villous. A serial section stained for LAIR-1 in the right hand panel shows that all trophoblast are negative. VM, villous mesencyme. [file figs3.jpg]
