# Supplementary material for: Genome-wide expression profile of first trimester villous and extravillous human trophoblast cells
Source: Placenta. 2011 Jan;32(1-3):33–43. doi: 10.1016/j.placenta.2010.10.010 (PMC3065343; doi:10.1016/j.placenta.2010.10.010)
Supplement: Suppl Table 1 — : GO biological processes over-represented in differences between the transcriptional profile of EVT and VT, with a significance threshold of p < 0.0025. [file mmc1.doc]

**Suppl Table 1: GO biological processes over-represented in differences between the transcriptional profile of EVT and VT, with a significance threshold of *p* < 0.0025.**

(A) GO Biological processes significantly UP-REGULATED in EVT compared to VT

| **P value** | **OR** | **ExpCt** | **Count** | **Size** | **Term** | **GOBPID** |
| --- | --- | --- | --- | --- | --- | --- |
| 0.00001 | 1.9 | 35.7 | 63 | 455 | cell motion | GO:0006928 |
| 0.0002 | 1.7 | 46.3 | 71 | 589 | immune response | GO:0006955 |
| 0.0004 | 1.5 | 77.1 | 106 | 996 | positive regulation of cellular process | GO:0048522 |
| 0.0005 | Inf | 0.2 | 3 | 3 | dermatan sulfate proteoglycan biosynthesis | GO:0050651 |
| 0.0006 | 6.4 | 1.6 | 7 | 20 | leukocyte adhesion | GO:0007159 |
| 0.0009 | 7.1 | 1.3 | 6 | 16 | glycosaminoglycan biosynthetic process | GO:0006024 |
| 0.0009 | 1.6 | 35.9 | 55 | 457 | positive regulation of developmental process | GO:0051094 |
| 0.0009 | 1.9 | 18.7 | 33 | 240 | enzyme linked receptor protein signaling | GO:0007167 |
| 0.0011 | 5.5 | 1.7 | 7 | 22 | activation of JUN kinase activity | GO:0007257 |
| 0.0011 | 15.7 | 0.5 | 4 | 7 | wound healing, spreading of epidermal cells | GO:0035313 |
| 0.0011 | 2.5 | 8.2 | 18 | 104 | response to virus | GO:0009615 |
| 0.0012 | 2.0 | 14.4 | 27 | 184 | regulation of response to stress | GO:0080134 |
| 0.0012 | 2.0 | 16.7 | 30 | 213 | induction of apoptosis | GO:0006917 |
| 0.0013 | 1.6 | 33.1 | 51 | 422 | protein kinase cascade | GO:0007243 |
| 0.0014 | 2.0 | 13.9 | 26 | 177 | regulation of anatomical structure morphogenesis | GO:0022603 |
| 0.0014 | 2.2 | 11.8 | 23 | 150 | positive regulation of kinase activity | GO:0033674 |
| 0.0015 | 8.4 | 0.9 | 5 | 12 | signal complex assembly | GO:0007172 |
| 0.0018 | 35.3 | 0.3 | 3 | 4 | regulation of transforming growth factor-beta2 | GO:0032909 |
| 0.0018 | 35.3 | 0.3 | 3 | 4 | positive regulation of hair follicle development | GO:0051798 |
| 0.0022 | 1.5 | 58.3 | 80 | 743 | regulation of cell communication | GO:0010646 |
| 0.0025 | 4.6 | 2.0 | 7 | 25 | positive regulation of cytoskeleton organization | GO:0051495 |
| 0.0025 | 1.8 | 19.0 | 32 | 242 | actin filament-based process | GO:0030029 |
| 0.0025 | 3.6 | 3.0 | 9 | 39 | wound healing | GO:0042060 |

(B) GO Biological processes significantly DOWN-REGULATED in EVT compared to VT

| **P value** | **OR** | **ExpCt** | **Count** | **Size** | **Term** | **GOBPID** |
| --- | --- | --- | --- | --- | --- | --- |
| 0.000010 | 1.9 | 34.0 | 60 | 446 | lipid metabolic process | GO:0006629 |
| 0.000014 | 1.8 | 41.2 | 69 | 533 | oxidation reduction | GO:0055114 |
| 0.0002 | 1.7 | 37.6 | 60 | 486 | oxoacid metabolic process | GO:0043436 |
| 0.0002 | 6.4 | 1.8 | 8 | 23 | fatty acid beta-oxidation | GO:0006635 |
| 0.0003 | 2.0 | 21.8 | 39 | 282 | generation of precursor metabolites and energy | GO:0006091 |
| 0.0005 | 23.9 | 0.5 | 4 | 6 | citrate metabolic process | GO:0006101 |
| 0.0005 | 2.1 | 15.2 | 29 | 196 | fatty acid metabolic process | GO:0006631 |
| 0.0005 | 2.2 | 13.8 | 27 | 178 | steroid metabolic process | GO:0008202 |
| 0.0006 | 1.8 | 28.1 | 46 | 363 | alcohol metabolic process | GO:0006066 |
| 0.0007 | 2.7 | 7.2 | 17 | 93 | carboxylic acid catabolic process | GO:0046395 |
| 0.0007 | 1.7 | 30.7 | 49 | 397 | response to organic substance | GO:0010033 |
| 0.0009 | 10.0 | 0.9 | 5 | 11 | regulation of skeletal muscle fiber development | GO:0048742 |
| 0.0009 | 2.3 | 10.7 | 22 | 138 | negative regulation of cell differentiation | GO:0045596 |
| 0.0010 | 4.8 | 2.2 | 8 | 28 | mesoderm formation | GO:0001707 |
| 0.0010 | 5.6 | 1.7 | 7 | 22 | cellular aldehyde metabolic process | GO:0006081 |
| 0.0010 | 16.0 | 0.5 | 4 | 7 | oxaloacetate metabolic process | GO:0006107 |
| 0.0011 | 2.7 | 6.8 | 16 | 88 | purine ribonucleoside triphosphate biosynthesis | GO:0009206 |
| 0.0011 | 3.8 | 3.2 | 10 | 42 | ion transmembrane transport | GO:0034220 |
| 0.0011 | 3.8 | 3.2 | 10 | 42 | lipid oxidation | GO:0034440 |
| 0.0012 | 2.6 | 6.9 | 16 | 89 | nucleoside triphosphate biosynthetic process | GO:0009142 |
| 0.0012 | 2.6 | 6.9 | 16 | 89 | ATP metabolic process | GO:0046034 |
| 0.0016 | 4.4 | 2.3 | 8 | 30 | regulation of pH | GO:0006885 |
| 0.0016 | 1.8 | 23.9 | 39 | 309 | lipid biosynthetic process | GO:0008610 |
| 0.0016 | 3.9 | 2.9 | 9 | 37 | ATP synthesis coupled proton transport | GO:0015986 |
| 0.0017 | 35.9 | 0.3 | 3 | 4 | taurine metabolic process | GO:0019530 |
| 0.0022 | 1.2 | 478.1 | 519 | 6452 | metabolic process | GO:0008152 |
| 0.0022 | 1.4 | 67.1 | 90 | 867 | cell proliferation | GO:0008283 |
| 0.0025 | 2.3 | 8.7 | 18 | 113 | ribonucleotide biosynthetic process | GO:0009260 |
