# Supplementary material for: Genome-wide expression profile of first trimester villous and extravillous human trophoblast cells
Source: Placenta. 2011 Jan;32(1-3):33–43. doi: 10.1016/j.placenta.2010.10.010 (PMC3065343; doi:10.1016/j.placenta.2010.10.010)
Supplement: Suppl Table 2 — : KEGG pathways over-represented in differences between the transcriptional profile of EVT and VT, with a significance threshold of p < 0.01. [file mmc2.doc]

**Suppl Table 2: KEGG pathways over-represented in differences between the transcriptional profile of EVT and VT, with a significance threshold of *p* < 0.01.**

(A) KEGG pathways significantly UP-REGULATED in EVT compared to VT

| **P value** | **OR** | **ExpCt** | **Count** | **Size** | **Term** | **KEGGID** |
| --- | --- | --- | --- | --- | --- | --- |
| 7 exp -6 | 2.2 | 24.1 | 47 | 311 | Pathways in cancer | 5200 |
| 2 exp -5 | 2.5 | 14.7 | 32 | 190 | Focal adhesion | 4510 |
| 0.0003 | 3.1 | 6.0 | 16 | 78 | Small cell lung cancer | 5222 |
| 0.0004 | 2.9 | 6.8 | 17 | 88 | Fc gamma R-mediated phagocytosis | 4666 |
| 0.0008 | 2.9 | 6.0 | 15 | 78 | ECM-receptor interaction | 4512 |
| 0.0014 | 2.9 | 5.1 | 13 | 66 | Pancreatic cancer | 5212 |
| 0.0015 | 2.4 | 8.4 | 18 | 108 | Leukocyte transendothelial migration | 4670 |
| 0.0017 | 2.5 | 7.1 | 16 | 92 | Toll-like receptor signaling pathway | 4620 |
| 0.0017 | 6.0 | 1.4 | 6 | 18 | Chondroitin sulfate biosynthesis | 532 |
| 0.0019 | 3.0 | 4.6 | 12 | 60 | RIG-I-like receptor signaling pathway | 4622 |
| 0.0020 | 1.9 | 15.7 | 28 | 203 | Regulation of actin cytoskeleton | 4810 |
| 0.0028 | 2.0 | 13.1 | 24 | 169 | Endocytosis | 4144 |
| 0.0040 | 1.7 | 19.6 | 32 | 253 | MAPK signaling pathway | 4010 |
| 0.0074 | 2.6 | 4.8 | 11 | 62 | Epithelial cell signaling in Helicobacter infection | 5120 |
| 0.0090 | 2.3 | 6.3 | 13 | 81 | TGF-beta signaling pathway | 4350 |

(B) KEGG pathways significantly DOWN-REGULATED in EVT compared to VT

| **P value** | **OR** | **ExpCt** | **Count** | **Size** | **Term** | **KEGGID** |
| --- | --- | --- | --- | --- | --- | --- |
| 2 exp -9 | 9.4 | 2.8 | 16 | 37 | Valine, leucine and isoleucine degradation | 280 |
| 2 exp -8 | 1.8 | 73.8 | 121 | 977 | Metabolic pathways | 1100 |
| 1 exp -6 | 8.5 | 2.0 | 11 | 27 | Propanoate metabolism | 640 |
| 7 exp -6 | 6.2 | 2.7 | 12 | 36 | Fatty acid metabolism | 71 |
| 9 exp -6 | 4.9 | 3.7 | 14 | 49 | Glutathione metabolism | 480 |
| 2 exp -5 | 18.4 | 0.8 | 6 | 10 | Reductive carboxylate cycle (CO2 fixation) | 720 |
| 4 exp -5 | 6.2 | 2.3 | 10 | 30 | Butanoate metabolism | 650 |
| 0.0004 | 4.8 | 2.4 | 9 | 32 | Cysteine and methionine metabolism | 270 |
| 0.0006 | 3.3 | 4.7 | 13 | 62 | Epithelial cell signaling in Helicobacter infection | 5120 |
| 0.0013 | 2.7 | 6.3 | 15 | 83 | ErbB signaling pathway | 4012 |
| 0.0017 | 3.8 | 2.9 | 9 | 38 | Pyruvate metabolism | 620 |
| 0.0031 | 2.8 | 4.9 | 12 | 65 | Biosynthesis of terpenoid alkaloids | 1066 |
| 0.0038 | 18.4 | 0.4 | 3 | 5 | Caprolactam degradation | 930 |
| 0.0039 | 6.1 | 1.1 | 5 | 15 | Steroid biosynthesis | 100 |
| 0.0055 | 2.7 | 4.6 | 11 | 61 | Biosynthesis of terpenoids and steroids | 1062 |
| 0.0062 | 2.7 | 4.7 | 11 | 62 | p53 signaling pathway | 4115 |
| 0.0082 | 2.3 | 6.2 | 13 | 82 | Biosynthesis of plant hormones | 1070 |
| 0.0091 | 4.7 | 1.4 | 5 | 18 | beta-Alanine metabolism | 410 |
| 0.0100 | 2.5 | 5.0 | 11 | 66 | Glycerophospholipid metabolism | 564 |
